# Potato Virus Y Emergence and Evolution from the Andes of South America to Become a Major Destructive Pathogen of Potato and Other Solanaceous Crops Worldwide

**DOI:** 10.3390/v12121430

**Published:** 2020-12-12

**Authors:** Lesley Torrance, Michael E. Talianksy

**Affiliations:** 1 The James Hutton Institute, Invergowrie, Dundee DD2 5DA, UK; michael.taliansky@hutton.ac.uk; 2 The School of Biology, University of St Andrews, North Haugh, St Andrews KY16 9ST, UK; 3 Shemyakin-Ovchinnikov Institute of Bioorganic Chemistry of the Russian Academy of Sciences, 117997 Moscow, Russia

**Keywords:** *Potyviruses*, whole genome sequencing, epidemiology, virus resistance, virus host interactions

## Abstract

The potato was introduced to Europe from the Andes of South America in the 16th century, and today it is grown worldwide; it is a nutritious staple food eaten by millions and underpins food security in many countries. Unknowingly, potato virus Y (PVY) was also introduced through trade in infected potato tubers, and it has become the most important viral pathogen of potato. Phylogenetic analysis has revealed the spread and emergence of strains of PVY, including strains causing economically important diseases in tobacco, tomato and pepper, and that the virus continues to evolve with the relatively recent emergence of new damaging recombinant strains. High-throughput, next-generation sequencing platforms provide powerful tools for detection, identification and surveillance of new PVY strains. Aphid vectors of PVY are expected to increase in incidence and abundance in a warmer climate, which will increase the risk of virus spread. Wider deployment of crop cultivars carrying virus resistance will be an important means of defence against infection. New cutting-edge biotechnological tools such as CRISPR and SIGS offer a means for rapid engineering of resistance in established cultivars. We conclude that in future, human activities and ingenuity should be brought to bear to control PVY and the emergence of new strains in key crops by increased focus on host resistance and factors driving virus evolution and spread.

## 1. Introduction

**Figure 1:**
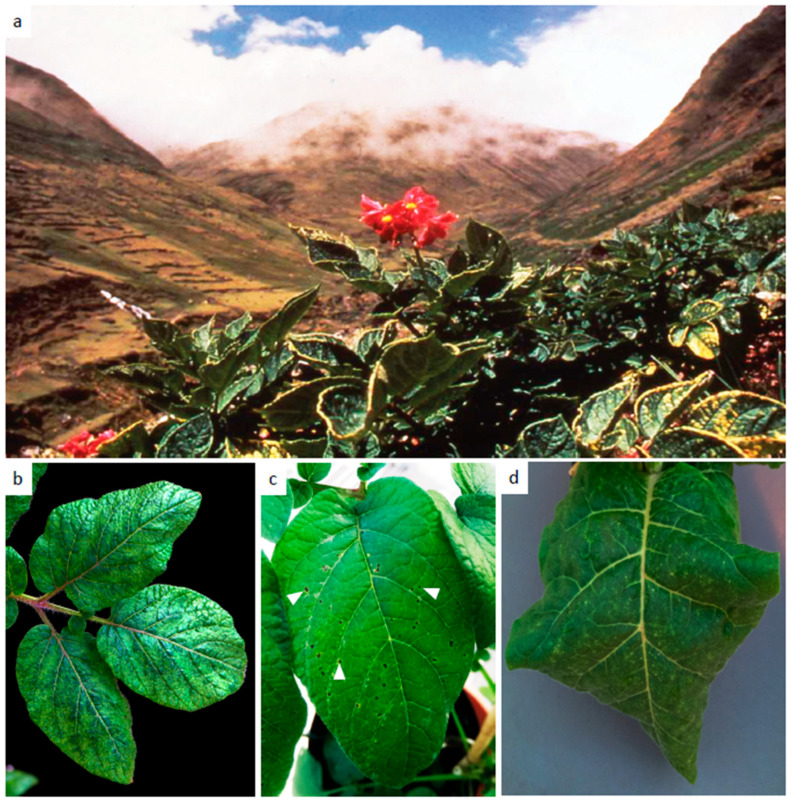
(**a**) Potato cultivation in the high Andes of Bolivia; (**b**) symptoms of O (ordinary) strain Potato virus Y (PVY^O^) on leaves of *Solanum tuberosum* Group Phureja (**c**) necrotic local lesions (indicated by white arrow heads) elicited by PVY^O^ in the inoculated leaves of potato cv Atlantic; (**d**) necrotic symptoms induced by N (necrotic) strain Potato virus Y (PVY^N^) in tobacco leaves.

Potato virus Y (PVY) is the type species of the genus *Potyvirus*, one of the largest groups of plant viruses, containing c. 160 species [1,2]. Potyviruses are transmitted by aphids and cause economically damaging diseases in crop plants. PVY exists as several strains and has become one of the most economically important pathogens of potato and the most important virus [3], and it occurs commonly wherever potatoes are grown. The Andean region of South America is the centre of origin of potato and many wild and domesticated species grow there (Figure 1a). After the discovery and colonization of the Americas by Europeans in the 16th century, tubers of one species of domesticated potato, *Solanum tuberosum*, were taken to Europe, and from there, over time, to the rest of the world [4,5]. Potato consumption grew in popularity in the 19th century, and it became an established staple food in many countries. Potatoes are vegetatively propagated, with progeny “seed” tubers being used to establish the next crop, and, unknown at the time, potato viruses were also transported with tubers. Potato viruses were first identified in the 1930s [6,7], and now more than 50 viruses affecting potato are known, although only a few, including PVY, cause economically important diseases [3]. Potato is the world’s third most important staple food crop and an important crop supporting food security in developing countries, where potato production now exceeds that in the developed world, and viruses are major constraints on potato production systems [3,8].

Human activity has played a major part in the spread of PVY from the South American Andes to the rest of the world, particularly through trade in plant material of unknown disease status. Since emerging from the Andean region, PVY has also become a major pathogen of tobacco and solanaceous vegetable crops [9,10,11,12,13]. This review highlights current knowledge of PVY population structure, epidemiology and economic impacts, mostly drawn from research on virus infections in potato. We believe that to effectively control the virus and prevent the emergence of new strains in key crops, future research should be strongly focused on host resistance and factors driving virus evolution and spread. Therefore, we also describe natural resistance mechanisms to PVY and how they can be modulated by rising temperatures under global warming. Modern biotechnology can play a role by developing genetically edited PVY-resistant crops as well as producing “vaccinated” plants by priming their antiviral defences through RNA silencing. This work is vital for the supply of nutritious food to a growing world population in disease-free and climate resilient, sustainable agricultural systems. 

## 2. PVY Population Structure

PVY was first identified by Smith [6], and several strains infecting potato were subsequently described in the early 20th century. Five major strain groups have now been recognised [14,15,16]. The first strains to be recognised were the O (ordinary) (Figure 1b), N (necrotic) and C (common) strains. These strains were characterised by biological properties and symptoms in potato hosts carrying strain-specific resistance genes (hypersensitive (HR) or *N* genes) [16,17,18]. The O, N and C strains were distinguished using potato cultivars carrying the genes *Ny* or *Nc*, which displayed necrotic spots on leaves when inoculated with O or C, respectively (Figure 1d) [17]. The N strain induces a systemic veinal necrosis in tobacco (Figure 1c) but generally no HR and only mild or no symptoms in potato leaves, and the C strain (PVY^C^) causes economically important diseases in other solanaceous crops including tobacco, tomato and pepper. In the 1980s, additional strains were reported where although mild or no symptoms were observed in leaves, some induced severe symptoms in potato tubers, such as potato tuber necrotic ringspot disease (PTNRD) [19,20]. Genome sequencing has revealed that the virus genomes of these isolates comprise sequences derived from O and N strains, these recombinant isolates included PVY^NTN^ and PVY^N-Wi^ [19,21,22,23]. The resistance gene *Nz* is effective against PVY^Z^ (shown to be the recombinant NTN). However, other recombinant strains such as PVY^N-Wi^ were not controlled by *Nz*, and they induce mild foliar symptoms with no PTNRD [18].

Phylogenetic analysis of 460 whole-genome sequences of PVY, collected from infected plants worldwide, showed that PVY originated in the Andes of South America, the centre of origin of the potato and where potatoes were first domesticated [14,15]. The date of origin (time to most recent common potyvirus ancestor) of the PVY population was estimated to be 1860 YBP. The maximum likelihood tree analysis largely supported the previous classification of strains based on host reactions, and five phylogroups were identified. Analysis of the nonrecombinant sequences produced three main lineages: the N phylogroup, which is widespread in South America and may only have spread to the rest of the world recently; the O phylogroup, isolates of which were found mostly from plants outside South America, and a branch of O, the C phylogroup, with no isolates found among South American samples. Moreover, the C isolates were often found in non-potato hosts, suggesting they may have diverged outside the Andes, possibly in Europe [15]. The analysis suggests that diversification and emergence of some of the current strains of PVY may have occurred outside South America, possibly in Europe. The first potato breeding programmes were based on a narrow genetic foundation of only a few introductions of potato, developing varieties through in-breeding and selection [4,14]. The introduction of new germplasm to combat susceptibility to late blight into potato breeding programmes in the mid-19th century probably led to PVY strain diversification, as this material also contained PVY resistance genes. Approximately half of the sequences analysed in the study by Fuentes et al. [15] comprised recombinants from N and O sequences and formed two further lineages, the R1 and R2 phylogroups. Recombinant isolates came to prominence in the 1980s and quickly spread, displacing other PVY strains in potato production systems, probably because they were uncontrolled by N genes and the mild foliar symptoms in many modern varieties enabled them to proliferate in seed crops because they escaped visual inspections [18]. Thus, since PVY was distributed from the Andes in potato tubers, the PVY population has evolved, with new strains emerging to infect non-potato hosts as well as overcome resistance of potato hosts, and human activity has been an important driver of this process [18].

## 3. Virus Epidemiology and Diagnostics

### 3.1. Natural Vectors 

Inoculum level and vector abundance are the most important factors in virus spread [24], and PVY is transmitted in nature by many different species of aphids in a nonpersistent manner [9]. The virus is acquired or inoculated within seconds through aphids probing and sampling the contents of epidermal cells with their stylets. Aphids will move to other plants if they decide not to feed (transferring virus as they go) and consequently, aphids that do not colonize potato are important vectors. Winged (alate) aphids can transmit virus between crops or between plants within a crop.

Many species of aphids can experimentally transmit PVY with varying efficiencies. *Myzus persicae* and *Macrosiphum euphorbiae* are efficient vectors that colonize potato, whereas noncolonizing cereal aphids such as *Rhopalosiphum padi* and *Sitobion avenae* that migrate in large numbers during the growing season are also important vectors. PVY incidence is correlated with aphid abundance early in the growing season [24], and so aphid incidence in the environment is often monitored. In the UK, the mean temperature in winter months (January and February) is considered a reliable guide to forecast the incidence and abundance of *M. persicae* [25]. Zhou et al. [26] found that winter temperature was the dominant factor affecting the phenology of five aphid species, including *M. persicae* and *S. avenae*, and that aphid migrations are 4–19 days earlier with each 1 °C increase in average winter temperature.

Application of insecticides is generally ineffective in decreasing PVY spread as they do not work fast enough to prevent aphids probing and transmitting the virus to a healthy plant. Mineral oils have been shown to be partially effective, but they must be applied frequently to ensure new growth is protected. The oils may interfere with aphid feeding or the interaction between virus particles and aphid stylets, but they can cause phytotoxicity and leaf marking that can obscure symptoms and interfere with visual inspection for virus. However, a combination of insecticide and mineral oil sprays applied weekly over the growing season was effective in decreasing PVY spread compared with unsprayed crops, and the effect was more pronounced when input seed had low levels of PVY (<1%) [24]. In another study, application of a straw mulch also significantly decreased PVY incidence by reducing aphid landing on the crop, whereas, in comparison, spraying mineral oil gave variable results [27]. Border crops to attract migrant aphids can also be useful as any aphid attracted to feed will lose the virus charge on probing. It was shown that covering pepper plants with 2 m wide plastic rowcovers immediately after transplanting protected them from aphid-borne virus and increased marketable yield [28].

It is known that the landscape structure affects the composition of arthropod communities within it [29]. A study of landscape composition, in terms of the relative amounts of cropland and unmanaged land, was conducted to investigate the effect on the incidence of PVY in potato crops [29]. They showed that more virus was found on farms located in areas with more crop land cover, whereas those farms in complex (unmanaged or nonagricultural) landscapes had much less virus (c. 30% vs. negligible, respectively). The results indicate that isolating potato production from other crops would decrease virus incidence.

### 3.2. Virus Sources

Common sources of infection are virus-infected potato plants, which can be infected tubers in the seed used to establish the crop, groundkeepers in neighbouring crops growing from tubers left in the soil or neighbouring crops being grown for consumption, which tend to have higher levels of virus than seed crops. Solanaceous weeds such as nightshades can also be reservoirs of infection [30]. PVY infection usually does not kill the susceptible plant outright, but virus will infect the progeny tubers, which in turn pass infection to the plants growing from these tubers; thus, infection quickly builds up in the tuber stock over subsequent field generations. In the most advanced potato production systems, classification programmes are in place to certify virus levels in seed stocks. These stipulate maximum virus levels, with the highest-grade seed having zero or a very small percentage of virus infection [18,31]. Virus levels in these stocks are usually estimated by visual inspections during the growing season. PVY strains that induce very mild or no symptoms in foliage can therefore be missed by visual inspection, and this has allowed virus levels to increase [18].

In the last 20–30 years, the recombinant strains of PVY have displaced the classical O and N strains and become the prevalent strains in European and US potato production systems [18,32,33]. This change in PVY strain composition is thought to have occurred because some commonly grown potato cultivars are tolerant to infection or contain strain-specific *N* resistance genes such as *Ny* (which was previously effective in controlling PVY^O^, the predominant strain infecting potato for many years) but are ineffective against recombinant strains. PVY^NTN^ was shown to overcome a type of host resistance that develops later in the growing season, called mature plant resistance (MPR) [34,35,36], infecting cv Maris Piper at the flowering stage when they were not susceptible to PVY^O^ [37] and indicating that PVY^NTN^ is capable of infecting plants later in the growing season. This is important in northerly countries such as Scotland that rely on plants developing MPR before aphid numbers increase.

### 3.3. Diagnostics 

PVY can be detected by serological and RNA-based techniques in samples of potato leaves or tubers [38]. Some potato seed classification schemes employ such laboratory-based tests to support visual inspections where symptoms are not obvious. These tests reveal the presence of known viruses. More recently, next-generation sequencing (NGS) techniques have been developed that can be applied to reveal unknown viruses and multiple infections in plants (the plant virome) [39]. Such NGS techniques can be deployed to sequence large numbers of virus genomes and compile phylogenies to discover recombinant genomes and monitor mutations and the incidence of new viruses, as well as monitor spread and genome changes in response to the deployment of disease-resistant cultivars. Obtaining information on the viromes of cultivated species and wild relatives at ecosystem boundaries can be used to monitor transmission from wild to cultivated hosts and to support predictive modelling tools and to evaluate land management practices [40]. NGS techniques provide valuable tools to monitor PVY strain emergence and evolution.

## 4. Economic Impacts of PVY

Estimating yield losses in potato due to PVY is complicated by several factors. For example, PVY can cause more severe symptoms in mixed infections with other viruses such as PVX. In addition, the time of infection can influence severity of disease, with young plants being more susceptible and displaying more severe symptoms when infected early in the season compared with plants where MPR has become established. The largest losses are when a crop is grown from infected seed tubers, and yield losses of 30%–64% have been reported [31,41]. 

In experiments with three potato cultivars, Russet Norkotah, Russet Burbank and Shepody, assessing yield from crops grown from seed lots containing different levels of PVY, it was found that for every 1% of PVY in the seed, the yield was decreased by 0.18 t/ha [42] and PVY decreased marketable yield and tuber size. An economic assessment estimated losses in the state of Idaho, USA, which produces approx. 7.1 M tonnes of potato, annually valued at $1 bn, to be $34 M (direct and indirect costs of production) [43]. It was estimated that 10% PVY infection in seed could decrease returns by $90–120 per acre depending on the market sector. 

PVY also causes major losses in peppers, tomato and tobacco [13]. For example, studies of the impact of PVY on field grown bell peppers showed a reduction of between 20% and 70% in the number of fruits per plant and the marketable yield depending on time of infection, with early infection most severely affecting the crop, and the marketable fruit yield was reduced up to 90% in mixed infections with CMV [44]. 

Potato production in low- and middle-income countries such as those in sub-Saharan Africa (SSA) is increasing, but the yields obtained are well below potential [45,46]. In SSA, production is confined to the rain-fed, cooler highland regions (>1500 m.a.s.l. (metres above sea level)) where potatoes are grown twice a year with little or no rotation; this leads to soil degradation and the build-up of soil-borne pests and diseases such as bacteria and nematodes. Farmers resort to cutting down forests to bring new land into cultivation to obtain better yields. Pest and disease surveys in the main potato-growing areas of Kenya have revealed that aphid vectors are abundant in the main growing seasons and that PVY (one of six viruses detected) is among the most predominant, being widespread in both seed and ware crops; recombinant strains PVY^NTN^ and PVY^N-Wi^ were also identified [47]. Although the potato yield gap is due to many factors, a major contributing factor is poor-quality seed and lack of access to seed tubers free of viruses [45,46,48]. Moreover, there was shown to be potential for a threefold increase in yield without expanding the potato production area [45]. Given the very high pest and disease pressure in the region, deployment of virus-resistant cultivars is vital to help control virus spread and improve productivity.

## 5. Host Resistance and Susceptibility

Breeding for virus resistance has not been a priority because seed certification systems were very effective in controlling virus. However, the mild foliar symptoms induced by some recombinant isolates have allowed these strains to avoid detection, meaning seed programmes based on visual inspection during the growing season are much less effective. Furthermore, given the problems and cost of PVY management through ineffective vector control measures, and with the incidence of vector aphids likely to increase due to increased survival over warmer winters, the deployment of resistant cultivars is considered to be the most effective and efficient means to control PVY [3].

Plant resistance to viruses is multifaceted. In the case of incompatible interactions, two main types of dominant host resistance against PVY are present in potato: strain-specific hypersensitive response (HR) or programmed cell death conferred by the *Ny* genes (as noted above) and extreme resistance (ER), conferred by the *Ry* genes. Potato plants containing various *Ry* genes are immune to PVY. ER is typically effective against a broad spectrum of PVY strains, and virus infection upon ER is limited to a few epidermal cells. Sources of *Ry* genes from several potato species are known [31,49,50]. To date, these ER genes have provided durable resistance against PVY.

The complex molecular mechanisms of HR and ER against PVY are described in detail in Baebler et al. [51]. Briefly, a first layer of innate immunity against viruses (as well as against other pathogens) is the recognition of pathogen-associated molecular patterns (PAMPs) by pattern recognition receptors (PRRs), which leads to PAMP-triggered immunity (PTI), where viral dsRNA plays the role of possible PAMPs [52]. A second layer of immune response occurs in plants carrying resistance (*R* or *N*) genes that employ effector-triggered immunity (ETI). In the case of HR, this involves the interaction between virus-derived effectors and host resistance R or N (mostly nucleotide-binding and leucine-rich repeat (NB-LRR) proteins that trigger a number of intracellular signalling events, which lead to disease resistance [51,53]. ER-mediated mechanisms are believed to be quite similar to those of HR. However, the ER conferred by the NB-LRR protein encoded by *Ry_sto_* does not depend on salicylic acid (SA) and requires EDS1 and NRG1 proteins. Moreover, in contrast to most HR-related resistance genes, *Ry_sto_* is not temperature sensitive [54].

In compatible interactions of potato with various viruses and PVY in particular, PTI-based SA-mediated signalling pathways also play important roles in determining resistance [55,56,57,58].

Another critical factor contributing to effective virus resistance is RNA interference (RNAi) or RNA silencing. RNAi is a sequence-specific mechanism degrading foreign nucleic acids and regulating endogenous gene expression. RNAi-based defence responses involve degradation of virus-derived double-stranded RNAs (dsRNAs) into small interfering RNAs (siRNAs), which in a complex with some plant proteins mediate the sequence-specific inactivation/degradation of viral RNAs [59,60,61]. However, during evolution, viruses have developed mechanisms to fight back against RNAi. For example, members of the genus *Potyvirus*, and PVY in particular, encode a silencing suppressor, which is the Helper Component—Proteinase HC-Pro protein [62]. Thus, the outcome of the PVY infection may be determined by a race between the activities of RNAi and silencing suppression. 

Remarkably, plant–virus interactions may also be regulated via the interplay between virus accumulation and plant methylation cycles (MTC) [63,64]. The MTC is functionally related to RNAi-based mechanisms, in which siRNAs (as major components of the RNAi-mediated defence response) are stabilized by MTC-associated transmethylation [65]. Plant DNA methylation, an epigenetic mechanism triggered by the MTC, has also been suggested to play an important role in modulating host responses to viruses by modifying functions of host genes and affecting gene expression [66,67]. Another factor released as a product of the MTC is ethylene, a phytohormone that, like other plant hormones, plays essential roles in plant responses to plant viruses [68]. Moreover, the potyviral HC-Pro silencing suppressor has been shown to physically interact with some components of the MTC [69], further confirming functional links between RNA silencing and MTC activities. 

Another form of host resistance is recessive resistance, for example, that conferred by the naturally existing resistant isoforms of eukaryotic translation initiation factors, eIF4E and eIFiso4E, has been shown to inhibit virus replication. Recessive resistance has been shown to be effective against PVY in vegetable crops such as tomato and pepper [70,71] and potato [72].

Finally, it is worth noting that some plant host factors may not only provide resistance against viruses but also may be hijacked by viruses for their own benefits [73]. For example, a key “signature” component of subnuclear Cajal bodies, coilin, is recruited by PVY to increase virus pathogenicity [73].

Thus, to survive in nature, PVY, like other plant viruses, has evolved virulence strategies to overcome host defences. The co-evolutionary arms race between PVY and the host has shaped current multifactorial defence and counter-defence mechanisms. Interestingly, several studies indicate the existence of inter-relationships between various antiviral defence (resistance) mechanisms. SA has been demonstrated to induce RDR1, which is a component of the antiviral RNAi pathway [74]. Moreover, some viral silencing suppressors may modulate SA-mediated resistance to several viruses, further confirming the existence of cross-talk between SA-mediated signalling and RNAi [75,76,77]. MTC-based defence pathways are also tightly inter-related with RNAi defence (through stabilisation of siRNAs, as noted above) and SA-mediated response (possibly through effect on the accumulation of another phytohormone, ethylene; see above). Thus, it is conceivable that different mechanisms of plant virus interactions (defence and counter-defence) form a specific integrated system that determines susceptibility/resistance to PVY in potato.

## 6. Effect of Environmental Stress: Temperature

Usually, potato, like other crops, is simultaneously exposed to various stresses, which modulate plant–virus interactions, and may cause further reductions in crop yield. Under climate change, temperature appears to be one of the critical environmental factors affecting plant growth and productivity. Potato is a cool-weather crop with optimal growth at temperatures ranging between 14 and 22 °C, above these temperatures, tuber yield is dramatically decreased [78]. From climate models it is expected that heat stress impacts on potato plants will become increasingly common, with potentially damaging effects on potato production over the world [79]. To cope with high temperatures, plants have evolved a variety of mitigation strategies that facilitate thermotolerance (for example, reference [80]).

It is also well established that heat stress can significantly but differentially affect plant–pathogen interactions via the modulation of host defence responses [58,81]. With regards to PVY, higher temperatures may result in positive or negative effect on the virus replication and spread. In incompatible interactions, most resistance genes, such as *Ny* from *S. sparsipilum* and *S. sucrense*, and *Ny-1* in potato cv. Rywal, confer resistance only at cooler temperatures (16–20 °C); at higher temperatures (24–28 °C) resistance does not develop, and PVY spreads systemically throughout the plant. In contrast, resistance to PVY^N^ expressed in *S. stoloniferum* (*Ry_sto_*) and *S. chacoense* (*Ry_chc_*) is effective at both low and elevated temperatures [82,83]. 

A differential impact of temperature on PVY accumulation has also been observed in compatible interactions: in cv. Chicago, elevated temperatures significantly increased susceptibility to PVY, whereas the effect of heat stress in cv. Gala was negligible [58]. However, mechanisms underlying thermo-sensitivity of defence responses in incompatible and compatible interactions are different. In incompatible interactions, temperature-dependent defence is seemingly due to temperature-sensitive conformational loss of function [84] in most resistance proteins, although products of *Ry_sto_* and possibly *Ry_chc_* resistance genes seem to be temperature-resistant and do not lose their activity at elevated temperatures [54]. In contrast, in compatible interactions, temperature-sensitive response might be controlled by the impact of heat stress on other regulatory components of the integrated defence system. 

Several effects of temperature on non-*R-* or *N-*gene-based host response mechanisms have been observed. First, it has been reported that RNAi-based defence is promoted by elevated temperatures, which may concomitantly attenuate development of the virus disease [85]. In contrast, RNAi suppression activity of the PVY suppressor HC-Pro is downregulated by higher temperatures, which could decrease defence and enhance PVY infection [86]. Second, activity of another anti-PVY defence factor, MTC, is significantly perturbed by rising temperatures in potato cv Chicago [64], resulting in a burst of the PVY infection. Third, SA is involved in both antiviral defence response and the regulation of heat shock protein (HSP) production and heat stress tolerance [87]. HSPs are known to take part in virus replication [88,89]. In turn, virus infections can modulate accumulation of HSPs [90]. 

Altogether, these findings suggest that responses to PVY infection and heat stress in potato have some common underlying mechanisms, which can be integrated in a network. Particular components of these networks may dominate in different virus–plant/cultivar combinations, allowing the defence responses under heat stress to be fine-tuned in a cultivar-specific manner. 

## 7. Engineering PVY Resistance in Potato

Genetic improvement for PVY resistance is essential for sustainable potato production. Conventional breeding to incorporate major resistance genes is still a useful approach to develop new cultivars, but the extreme heterozygosity and complex genetics of potato mean that even with new genetic marker technologies, it is a time-consuming and laborious process [4,91]. Another well-established approach to develop PVY resistance is based on transgenic technology. Transgenic potato plants overexpressing PVY-derived coat protein, PVY-specific dsRNA (for RNAi) or modified plant eIF4E all demonstrated a high level of resistance. However, the commercial development of transgenic potato or other vegetable crops is constrained by regulations surrounding the release of GM plants and negative public perception. Both these approaches have been extensively discussed in a number of previous reviews [92,93]. In this review, we will focus on two technologies that have emerged in the past decade, namely CRISPR/Cas (clustered regularly interspaced short palindromic repeats/CRISPR-associated genes) and spray-induced gene silencing (SIGS), which provide new methods for improvement of PVY resistance that may be less contentious. 

CRISPR/Cas is a prokaryotic adaptive immune system that has been reprogrammed into a precise and powerful tool for precise gene targeting [94]. In this system, Cas9 DNA exonuclease is guided by a short RNA (sgRNA) that defines the genomic DNA target to be modified (inducing deletions, insertions or replacements). Other types of exonucleases such as Cas13 or FnCas9 can target RNA molecules. The CRISPR/Cas system has now been extensively exploited to generate plant virus resistance. This has been achieved either by direct inhibition of viral RNAs/DNAs or by introduction of mutations into host plant “susceptibility” genes [95]. Both approaches have been successfully used to derive resistance to PVY. In the first approach, Zhan et al. [96] engineered resistance by directly targeting the PVY *P3*, *CI*, *NIb* and *CP* genes in transgenic potato expressing Cas13 and gene-specific sgRNAs. 

In another approach, Makhotenko et al. [97] used the CRISPR/Cas9 tool to generate PVY resistance by targeted mutagenesis of the coilin gene in potato. An important aspect of this work is that the authors developed a new technology to achieve transgene-free genome edits and avoid the use of DNA at all. For this purpose, they delivered DNA-free CRISPR/Cas9 RNP complex pre-assembled from Cas9 and sgRNA into apical meristematic tissues of potato. 

Spray-induced gene silencing (SIGS) is another RNAi-based genome technology for targeting various pests and pathogens (including viruses). Application of exogenous dsRNAs by spraying plants has been successfully exploited to induce resistance to different viruses in a wide range of crops [95]. SIGS technology for disease control appears to be potentially sustainable and environmentally friendly and could be used to protect potato from PVY.

## 8. Conclusions and Perspectives

Human activity has been responsible for the spread of PVY, with consequent severe losses in yields of potato and other solanaceous crops. PVY phylogeny suggests that the PVY population continues to evolve, with new strains emerging that infect new, non-potato hosts as well as defeating host resistance responses. New NGS technologies provide a powerful means to support modelling to identify the emergence and spread of new strains. PVY is transmitted by many species of aphids and can be acquired and transferred from plant to plant in seconds by the aphid stylet probing of plant cells. Aphid vector control methods are only partially effective, and the widespread use of agri-chemicals for their control is environmentally undesirable. Aphid populations are predicted to increase in size and migrate earlier in the growing seasons in warming environments, increasing the risk of virus spread. Therefore, host resistance is concluded to be the most economically effective and efficient means of control in sustainable potato production systems. Some of the known PVY resistance genes can be ineffective in warmer environments; recent research has produced much knowledge of virus–host interactions in response to abiotic stresses to aid understanding of host resistance mechanisms, however, more work is needed on how virus resistance is affected by temperature. New, advanced biotechnological tools such as CRISPR and SIGS offer huge potential to introduce virus resistance into established cultivars, thus enabling rapid development and deployment of these enhanced cultivars for efficient and sustainable crop production. However, gene target identification is an important challenging step. Another challenge is to develop efficient technologies for delivery of biomolecules such as dsRNA into intact plant cells. The production of varieties using long-established transgenic technology is contentious in some countries; the new CRISPR and SIGS technologies have the advantage of enabling modifications without the introduction of additional transgene DNA so may be more publicly acceptable, however, this remains to be resolved.
